# The role of plant–mycorrhizal mutualisms in deterring plant invasions: Insights from an individual‐based model

**DOI:** 10.1002/ece3.4892

**Published:** 2019-01-28

**Authors:** Matthew A. McCary, Moira Zellner, David H. Wise

**Affiliations:** ^1^ Department of Biological Sciences University of Illinois Chicago Illinois; ^2^ Institute for Environmental Science and Policy University of Illinois Chicago Illinois; ^3^ Department of Urban Planning and Policy University of Illinois Chicago Illinois; ^4^ Present address: Department of Entomology University of Wisconsin Madison Wisconsin

**Keywords:** competition, individual‐based model, intermediate mutualist‐strength hypothesis, invasion resistance, invasive plants, mutualism, mutualism disruption, mycorrhizal fungi

## Abstract

Understanding the factors that determine invasion success for non‐native plants is crucial for maintaining global biodiversity and ecosystem functioning. One hypothesized mechanism by which many exotic plants can become invasive is through the disruption of key plant–mycorrhizal mutualisms, yet few studies have investigated how these disruptions can lead to invader success. We present an individual‐based model to examine how mutualism strengths between a native plant (*Impatiens capensis*) and mycorrhizal fungus can influence invasion success for a widespread plant invader, *Alliaria petiolata* (garlic mustard). Two questions were investigated as follows: (a) How does the strength of the mutualism between the native *I. capensis* and a mycorrhizal fungus affect resistance (i.e., native plant maintaining >60% of final equilibrium plant density) to garlic mustard invasion? (b) Is there a non‐linear relationship between initial garlic mustard density and invasiveness (i.e., garlic mustard representing >60% of final equilibrium plant density)? Our findings indicate that either low (i.e., facultative) or high (i.e., obligate) mutualism strengths between the native plant and mycorrhizal fungus were more likely to lead to garlic mustard invasiveness than intermediate levels, which resulted in higher resistance to garlic mustard invasion. Intermediate mutualism strengths allowed *I. capensis* to take advantage of increased fitness when the fungus was present but remained competitive enough to sustain high numbers without the fungus. Though strong mutualisms had the highest fitness without the invader, they proved most susceptible to invasion because the loss of the mycorrhizal fungus resulted in a reproductive output too low to compete with garlic mustard. Weak mutualisms were more competitive than strong mutualisms but still led to garlic mustard invasion. Furthermore, we found that under intermediate mutualism strengths, the initial density of garlic mustard (as a proxy for different levels of plant invasion) did not influence its invasion success, as high initial densities of garlic mustard did not lead to it becoming dominant. Our results indicate that plants that form weak or strong mutualisms with mycorrhizal fungi are most vulnerable to invasion, whereas intermediate mutualisms provide the highest resistance to an allelopathic invader.

## 1 INTRODUCTION

The introduction of invasive plants into local ecosystems is one of the most significant threats to natural plant and animal populations (Moser et al., 2009; Vilá et al., 2011). Invasive plants modify ecosystems by changing vegetation structure and productivity (Asner et al., 2008), resulting in habitat and biodiversity loss. Invasive plants also disrupt the functioning and energy flow of a system via changes to food‐web dynamics (McCary, Mores, Farfan, & Wise, 2016; Smith‐Ramesh, Moore, & Schmitz, 2017), soil chemistry and nutrient availability (Ehrenfeld, Kourtev, & Huang, 2001), and disturbance regimes (Brooks et al., 2004; Mack & D'Antonio, 1998). Thus, a current goal in ecology is to understand which factors most strongly influence invasion success for non‐native plants (Hejda, Chytrý, Pergl, & Pyšek, 2015; Higgins & Richardson, 2014; Moravcová, Pyšek, Jarošík, & Pergl, 2015).

One proposed mechanism by which plant invaders can negatively impact natives is through their disruption of key mutualisms between native plants and mycorrhizal fungi (Grove, Haubensak, Gehring, & Parker, 2017; Hale & Kalisz, 2012; Hale, Tonsor, & Kalisz, 2011). Mutualism disruption is an extension of the novel weapons hypothesis (Callaway et al., 2008; Callaway & Ridenour, 2004) and may explain why some invasive plants are so pervasive. Mycorrhizal fungi form important symbioses with most plants by increasing the root's ability to absorb nutrients and water available in the soil (Smith, Facelli, Pope, & Smith, 2010). The fungus, in return, benefits by receiving photosynthetically derived carbohydrates from the plant (Parniske, 2008). Because 80%–90% of all plant species associate with mycorrhizal fungi in some manner (Smith & Read, 2010), a non‐native plant that interferes with this interaction will likely have a competitive advantage over most native plants. For instance, several invasive plants can indirectly limit seedling and mature‐plant growth by releasing toxic fungicidal chemicals (i.e., allelochemicals) into the soil (Brouwer, Hale, & Kalisz, 2015; Lankau, 2012), which reduce fungal densities, thereby weakening the mutualism and lowering the per‐capita growth rate of the native plant (Hale, Lapointe, & Kalisz, 2016).

Although prior research has indicated that plant–mycorrhizal disruptions can lead to invasion success for many plant invaders, previous studies have ignored how the strength of the mutualism affects resistance to invasion. Plant–mycorrhizal mutualisms function on a continuum from facultative to obligate (Johnson & Graham, 2013). The strength of this mutualistic interaction is influenced by several factors, including the plant species, local environmental conditions, and the presence/absence of parasitic microorganisms (Hale & Kalisz, 2012; Hoeksema et al., 2010). For example, in high‐nitrate soils, the mutualism may become parasitic, with the fungus removing photosynthates without providing any benefits to the plant (Treseder & Allen, 2002). In contrast, in low‐phosphorus environments, the interaction typically functions as a mutualism in which both partners benefit (Ji & Bever, 2016). Therefore, to fully understand how plant–mycorrhizal disruptions influence invasive success, we need to evaluate the disruption across a gradient of mutualism interaction strengths. The degree to which a given plant species depends on this mutualism should determine its susceptibility to plant invaders that release fungicides; however, creating a natural gradient to test this prediction under field or laboratory conditions presents many logistical challenges.

One approach to answering this important, yet experimentally challenging, question is to use an individual‐based model (IBM). IBMs simulate individual components in a complex system, based on simple rules and treating individuals as unique and discrete entities (Grimm & Railsback, 2005; Railsback & Grimm, 2012). In IBMs, individuals (also termed “agents”) simultaneously interact with one another and with the environment (Railsback & Grimm, 2012). To facilitate realistic representations of complex ecological systems, agents can adapt to altered environmental conditions and therefore influence future generations of agents (Wilensky & Rand, 2015). Thus, IBMs provide an ideal framework to examine how a continuum of interaction strengths might affect the success of a plant invader.

In this study, we present an IBM that models how mutualism strengths can influence the invasiveness of an allelopathic plant invader. We used garlic mustard (*Alliaria petiolata)* as the example because it is the best‐documented case of an invasive plant indirectly affecting the growth and abundance of native plants by suppressing mycorrhizal fungi (Brouwer et al., 2015; Hale et al., 2011). It is a pervasive invader with the potential to create dense monocultures in forest understories and has been a prominent challenge for land managers since it was first introduced into North America in the 1860s (Anderson, Dhillion, & Kelley, 1996; Meekins & McCarthy, 1999; Nuzzo, 1999). Garlic mustard releases glucosinolates that disrupt the plant–mycorrhizal mutualism by reducing the abundance of both arbuscular (Roberts & Anderson, 2001; Stinson et al., 2006) and ectomycorrhizal fungi (Wolfe, Rodgers, Stinson, & Pringle, 2008).

To better understand how disruptions to plant–mycorrhizal mutualisms could explain variation in garlic mustard's invasiveness, we developed an IBM to answer two questions: (a) How might the strength of the mutualism between a native plant (*Impatiens capensis*) and a mycorrhizal fungus affect the plant's resistance to garlic mustard invasion? (b) Can there be a non‐linear relationship between initial garlic mustard density and establishment? We hypothesized that strong mutualisms, that is, higher nutrient exchanges between the mycorrhizal fungus and the native plant, would exhibit the highest resistance to plant invasion. Increased nutrient exchanges should ultimately lead to higher fecundity for both fungi and plants, resulting in lower garlic mustard densities. Furthermore, we hypothesized that there would be a starting density that garlic mustard must reach before becoming invasive, which we defined as garlic mustard eventually representing more than 60% of the total plant cover.

## 2 MATERIALS AND METHODS

### 2.1 Model description

Here we outline the main characteristics of the model; the full description using the Overview, Design concepts, and Details (ODD) protocol (Grimm et al., 2010, 2006) is provided in Supporting Information Appendix S1. The purpose of this model was to examine how disruptions to plant–mycorrhizal mutualisms can explain invasion success for garlic mustard (*A. petiolata*)—the widespread North American plant invader of forest ecosystems. To accomplish this goal, we designed a spatially explicit IBM using NetLogo version 5.3.1 (Wilensky, 1999). The spatial framework depicted a 200 × 200 two‐dimensional woodland understory, with discrete time steps representing a 10‐day period within the growing season. There were three agents in this model: (a) a native annual of North American *I. capensis *(hereafter referred to as the “native plant”); (b) a “generic” arbuscular mycorrhizal fungus that forms a mutualistic association with the native plant; and (c) the non‐native invasive garlic mustard.

Mycorrhizal “nodes” are randomly distributed within the model environment, with each node being able to grow (or lose) hyphae at a certain rate (***F***) depending on how much photosynthetically derived carbon (***C***) is available, which is directly linked to the presence of the native plant. The mycorrhizal symbiosis is modeled by the provision that the native plant releases ***C*** at a rate depending upon the strength of the mutualism and whether a fungus is present. When the fungus is present (***C*_r_**), native plants will have higher nutrient‐absorption rates (***E***), thus leading to increased photosynthetic rates and fecundity (i.e., offspring produced per plant). As the two species engage in nutrient exchanges (the fungus gets ***C*_r_**, and the native plant receives ***E*** [e.g., nitrogen, phosphorous, and potassium] to support photosynthesis and growth), the fungus will assimilate available ***C*** at a higher rate (***C*_m_**) and reproduce quicker, and the plant will produce more offspring in the next cohort (***N*_H_**). In contrast, if the two are not in the same patch, photosynthetic output (***e***) for the native plant is lower and therefore produces fewer offspring (***N*_L_**), and the mycorrhizal fungus dies after approximately three time steps. *I. capensis* is an annual herb (Dudley & Schmitt, 1996), so we programmed for the native plant to set seed and die after one growing season (i.e., 10 time steps in the model [representing ~100 days]; Abrahamson & Hershey, 1977). Given the different time scales in which the native plant and mycorrhizal fungus function (the fungus grows and turns over much faster than plants), this programming of time steps allowed for meaningful interactions to occur between the two species.

Because garlic mustard is a known disrupter of plant–mycorrhizal mutualisms, we modeled it to kill the fungus within a specified patch radius (***A***). Garlic mustard does not form mutualisms with any known fungi; thus, we programmed the plant invader to produce a constant rate of offspring without any nutrient exchanges with the soil or mycorrhizal fungus. Garlic mustard dies and sets seed (***R***) within a given radius in decimeters (***D***) after 20 time steps (~200 days), representing a biennial life cycle. For this IBM, we chose a range of initial densities of garlic mustard (***I***) to represent the early stages of plant invasion (Anderson et al., 1996; Nuzzo, 1999).

**Figure 1:**
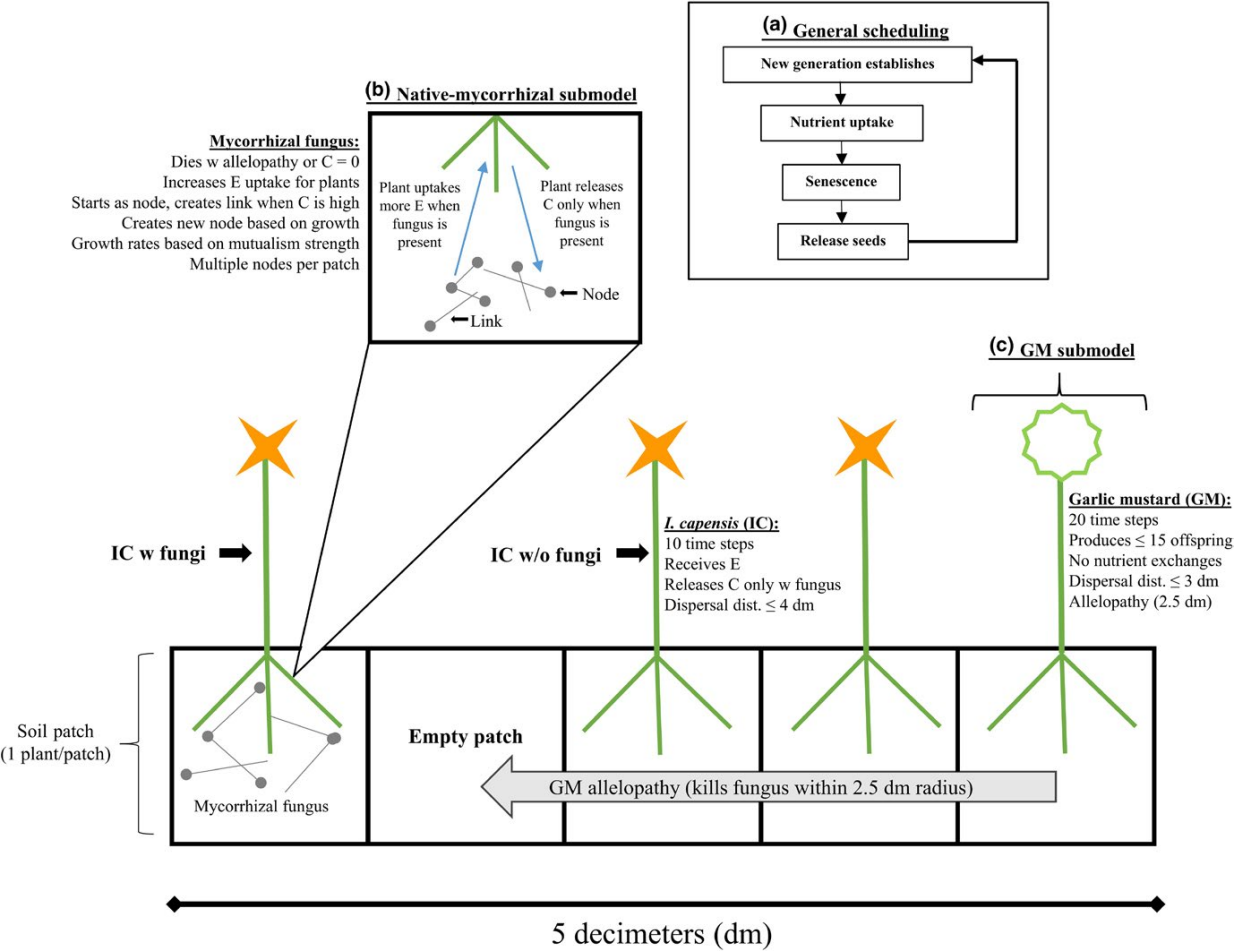
A schematic of the model structure. (a) Scheduling of major events (top, middle). (b) Native plant and mycorrhizal fungus sub‐model (top, left). (c) Garlic mustard submodels (bottom, right). Yellow flowers represent *Impatiens capensis*; white flowers, garlic mustard

The main interaction between the native plant and garlic mustard is competition for space (Figure 1). In the model, no two plants of either the same or the different species can occupy the same patch, leading to space limitation in the production of offspring. This simplification allows for a one‐to‐one competition for space, making it easier to compare differences in population sizes. Native plants that acquire a symbiosis with a mycorrhizal fungus will gain a competitive advantage over garlic mustard by producing more offspring at the end of the season. This process is synergistic: As more fungal connections are made with the native plant (i.e., through an increase in hyphae), both the native plant and the mycorrhizal fungus will colonize more patches. However, garlic mustard can indirectly affect the native plant by killing the fungus, thereby slowing its growth and favoring garlic mustard. For model simplicity, we did not model a cost for being a mutualist for either the native plant or the mycorrhizal fungus.

Because the main focus of this model was to understand how mutualism strengths can mediate the invasiveness of an allelopathic plant invader, we structured our model to be simple and computationally tractable. Here we outline the simple assumptions in our individual‐based model. Although we used *I. capensis* and garlic mustard as real‐world examples, we did not model every characteristic associated with the two plants. One simplification is that we ignored the different phenology and life stages of the two plants. We programmed the plants to emerge as mature plants where they uptake nutrients, senesce, release seeds, and produce “offspring.” Offspring in our model encompasses all phases of a plant's life cycle, including germination, seedling survival, and adult mortality. Another simplification is that we did not model a seed bank for either the native plant or the garlic mustard. Modeling a seed bank would have made the model more realistic but is unnecessary for determining how disruptions to plant–mycorrhizal mutualisms can contribute to invader success. One last simplification is that we did not model the potential legacy effects of garlic mustard's allelopathy. While we recognize that legacy effects will undoubtedly affect the dominance of an allelopathic invader, it is a different question than how mutualism strengths can influence the invasiveness of a plant invader.

### 2.2 Parameterization and calibration

The parameter values of each agent were largely based on field and laboratory experiments, but we also used pattern‐oriented modeling (POM; Grimm et al., 1996) to parameterize unknown values when data were scarce. Pattern‐oriented modeling aims to reproduce the observed patterns that occur in nature at multiple spatial and temporal scales (Grimm et al., 2005; Wiegand, Jeltsch, Hanski, & Grimm, 2003). Submodels of the native plant–mycorrhizal mutualism and garlic mustard invasion were developed and evaluated based on how well they reproduced natural patterns (plant cover, mutualism strengths, and equilibrium densities). Model outputs represented realistic population sizes (100–350 plants per m^2^; Steets, Knight, & Ashman, 2007) and growth rates (Kalisz, Spigler, & Horvitz, 2014) for both garlic mustard and *I. capensis*. Refer to Table 1 for parameters that were obtained from laboratory and/or field experiments, and the values parameterized using POM.

**Table 1:** Model parameters with their respective description and factors

Parameters	Description (units)	Values			Sources
		Weak	Intermediate	Strong	
Native plant					
***E***	Photosynthetic rate with fungus present (energy units)	0.45	0.75	1	POM
***e***	Photosynthetic rate without fungus (energy units)	0.4	0.3	0.01	POM
***C*_r_**	C molecules released with fungus (C units time step^−1^)	4.5	9	15	POM
***N*_H_**	No. offspring produced when energy is high (per plant)	≤6	≤10	≤20	POM
***N*_L_**	No. offspring produced when energy is low (per plant)	≤4	≤5	≤3	POM
Fungus					
***F***	Fungal growth rate with native plant (mm time step^−1^)	0.01	0.15	0.30	POM
***C*_m_**	C assimilated from plant (µmol C time step^−1^)	1.5	2.5	4	Hobbie, 2006
Patches					
***C***	Initial patch C value	≤4	≤8	≤9	—

		Range			
		Default	Min	Max	
Garlic mustard					
***I***	Starting no. GM plants (per 400 m^2^)	500	1	1,000	Anderson et al., 1996
***R***	No. offspring GM can produce (dm^2^)	15	1	15	Anderson et al., 1996
***D***	Distance GM can produce new plants (dm^2^)	3	1	3	Nuzzo, 1999
***A***	Patch radius GM can suppress fungus (dm)	2.5	—	—	Wolfe et al., 2008

		Fixed values			
Initialization					
Native density	Starting no. native plants (per 400 m^2^)	10,000			Steets & Ashman, 2010
Native dispersal	Patch distance a native plant can sprout new plants (dm)	1			POM
Invasive energy	Invasive energy (generic units)	1			POM
Fungus nodes	Node initial abundance (400 m^2^)	10,000			POM
Node size	Maximal node biomass (mm)	0.3			POM
Hyphae	Maximal extra‐radical hyphae size (links) (mm)	0.3			POM

Notes

“—“: no data or source is available; dm: decimeter; m: meter; mm: millimeter; mol: one mole unit; POM: pattern‐oriented modeling.

The three levels of “Values” refer to the relative strength of the mutualistic interaction.

We used *I. capensis* as the native competitor because (a) garlic mustard is known to invade areas in which *I. capensis* naturally occurs, (b) it forms a symbiotic relationship with mycorrhizal fungi, and (c) other studies have used this plant as the native comparison to garlic mustard (e.g., Meekins & McCarthy, 1999; Wolfe et al., 2008). In the presence of mycorrhizal fungi, *I. capensis* has a high reproductive rate due to high rates of soil‐nutrient uptake; its reproductive output is much lower when the mutualism is not present (Cipollini, McClain, & Cipollini, 2008). We simulated *I. capensis* with a 1‐year life cycle, with the ability to produce multiple plants once a certain photosynthetic‐energy threshold is reached, which is influenced by the presence of a mycorrhizal fungus. We programmed *I. capensis *to release generic photosynthetic ***C*** values, which serves as the main source of energy for the mycorrhizal fungus. *I. capensis* is a dehiscent ballistic disperser (Hayashi, Feilich, & Ellerby, 2009), that is, the seed pod explodes to release seeds that travel a short distance (3–5 decimeter (dm); Schmitt, Ehrhardt, & Swartz, 1985). In the model, plants can reproduce up to a certain distance, which was done using a random draw between the minimum and maximum distances assigned for each plant. The direction in which a plant can produce offspring was also random.

Studies to date have estimated that mycorrhizal fungi assimilate photosynthetically fixed carbon from a native plant's root system at rates of 4%–30% (Hobbie, 2006; Kaschuk, Kuyper, Leffelaar, Hungria, & Giller, 2009); we simulated ***C*** to be assimilated within this range (Table 1). We also modeled the mycorrhizal fungus to grow as a function of available photosynthetic ***C***: the more photosynthetic ***C*** from the plant, the higher the growth rate of the mycorrhizal fungus. When no plant was available, the fungus will consume its available ***C*** reserves and die if a native plant does not colonize the patch after approximately three time steps. The default ***C*** assimilation and growth rates are based on generic units.

Garlic mustard limits the growth of soil fungi by releasing allelochemicals that can diffuse up to 2.5 dm in the soil (Wolfe et al., 2008). Garlic mustard, which has a 2‐year life cycle and can produce hundreds of seedlings per plant in the second year (Anderson et al., 1996; Roberts & Anderson, 2001), was modeled to produce up to 15 adult offspring per plant. The above traits of garlic mustard were parameterized, calibrated, and implemented in the model (Table 1).

### 2.3 Sensitivity analysis

We employed a modified version of the elementary effects method (i.e., the Morris screening method; Morris, 1991, Campolongo, Cariboni, & Saltelli, 2007, Thiele, Kurth, & Grimm, 2014) to rank the parameters according to their influence on abundances of fungal nodes, native plants, and garlic mustard. Seven model parameters were varied across five levels using values from model simulations; thus, 35 parameter sets were analyzed, with each being replicated five times. The sensitivity of the model to each parameter was evaluated using the mean absolute value of elementary effects (μ*), which measures the overall impact of a parameter on the output. The standard deviation of the elementary effects values (*σ*) was also used to measure higher‐order effects (nonlinear and/or interaction effects; Campolongo et al., 2007). As output, we show groups of values associated with the three main agents of interest in the model—native plant, mycorrhizal fungus, and garlic mustard.

### 2.4 Simulation experiments

To simulate different levels of mutualism strengths (Question 1), we constructed three model versions: a weak, intermediate, or strong mutualistic interaction between the fungus and native plant (Table 1). All three models were identical except that the rates of nutrient exchange, mycorrhizal growth, and reproductive outputs were set to correspond to the level of mutualism strength (i.e., low rates of nutrient exchange and growth, when both species are present, represent a weak mutualism; medium rates of exchange and growth defined an intermediate mutualism, and so forth). For weak mutualisms, *I. capensis* could produce up to six offspring per plant when the fungus was present, or up to four with no fungus. For intermediate and strong mutualisms, the native plant was able to produce up to 10 and 20 offspring per plant when the fungus was present, respectively, and five and three when the fungus was not present, respectively. Once the three different levels of mutualism strength had been established, we simulated the introduction of garlic mustard under different initial densities: one individual per 400 m^2^ (0% plant cover), 100 individuals per 400 m^2^ (1% plant cover), 500 per 400 m^2^ (5% plant cover), and 1,000 per 400 m^2^ (~10% plant cover). These values were chosen to represent early stages of plant invasion (Anderson et al., 1996; Nuzzo, 1999).

After the three levels of mutualism strength had been evaluated against different initial densities of garlic mustard invasion, we used the level of mutualism with the highest invader resistance (i.e., final garlic mustard numbers <60% of plant density) to examine whether there was an initial population size that guarantees successful invasion by garlic mustard (Question 2). We extended the initial population sizes of garlic mustard to densities of 2,000, 3,500, and 6,000 per 400 m^2^_._ After 100 time steps for each scenario, we recorded the number of mycorrhizal fungal nodes and the population sizes of both native plant and garlic mustard. Each model simulation was replicated ten times.

### 2.5 Statistical analyses

To evaluate the primary questions, we first evaluated line graphs with standard errors of changing densities of the three agents as a function of initial garlic mustard density. We also used analysis of variance (ANOVA), followed by Tukey's HSD post hoc comparisons, to assess the impact of differences in mutualism strength on abundances of the native plant, fungal nodes, and garlic mustard. To examine the temporal dynamics of the three agents, we ran a separate set of 10 simulations for the three mutualism strengths and provided the average density for each of the 100 time steps. To understand how each parameter influenced equilibrium population sizes for the native plant, fungus, and garlic mustard under the most resistant mutualism strength, we used bivariate heat maps. All statistical analyses were performed with the R Statistical Computing language (R Development Core Team, 2016); the sensitivity analysis was conducted using the "sensitivity" package in R (Thiele et al., 2014).

## 3 RESULTS

### 3.1 Sensitivity analysis

**Figure 2:**
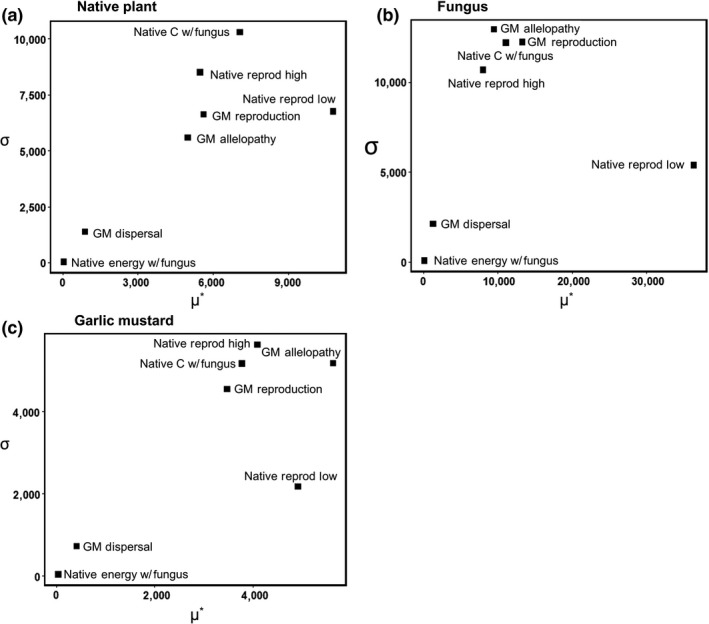
Sensitivity to variation in model parameters of model outputs for the three agents. (a) Mean density of the native plant. (b) Mean density of the fungus. (c) Garlic mustard's mean density. µ* = mean of the absolute values of elementary effects; *σ* = standard deviation of the elementary effects

Different parameters were most influential on equilibrium densities of native plant, fungus, and garlic mustard (Figure 2). Equilibrium density of the native plant was most sensitive to the amount of ***C*** it released when the fungus was present, which had high values for both µ* (the mean of the absolute values of elementary effects) and *σ* (the standard deviation of the elementary effects). No other parameters were nearly as influential in determining native plant density except for native reproduction when the fungus was present (Figure 2a). For equilibrium fungal density, garlic mustard allelopathy, garlic mustard reproduction, and the amount of ***C*** released when the native was present were all influential (Figure 2b). Garlic mustard density was most affected by its allelopathy and rate of reproduction, native rate of reproduction when the fungus is present, and how much ***C*** is released from the native plant when the fungus is present (Figure 2c). Most of the highly influential (i.e., most sensitive) parameters were also involved in higher‐order effects (Supporting Information Appendix S2); that is, they had non‐linear interactions, and their impact depended strongly upon the value of other input factors. High values of both µ* and *σ* indicate an interactive effect on equilibrium density of the agent, whereas high µ* with low *σ* signifies the absence of interactions (Thiele et al., 2014).

Simply examining the sensitivity of parameters one‐by‐one does not adequately answer the two central questions, for two reasons. First, as mentioned above, the effects of several parameters were highly non‐additive. Second, a major goal of the IBM was to examine the influence of the strength of the fungal‐native plant mutualism on the native plant's resistance to invasion by garlic mustard. Since the three levels of mutualism strength were defined by unique combinations of three levels of five mutualism parameters (***E***, ***e***, ***C*_r_**, ***F***, and ***C*_m_**), it is the behavior of the model under these restricted sets of parameter values that is of primary interest. Thus, the best way to use this IBM to answer the main research objectives is through simulation experiments focused first on varying mutualism strength.

### 3.2 Simulation experiments

#### 3.2.1 Mutualism strength

The strength of the mutualism between the native plant and fungus played a major role in determining garlic mustard's invasion success. Several patterns in the simulations reveal that a native plant with a mutualism of intermediate strength was most resistant to invasion by garlic mustard.

**Figure 3:**
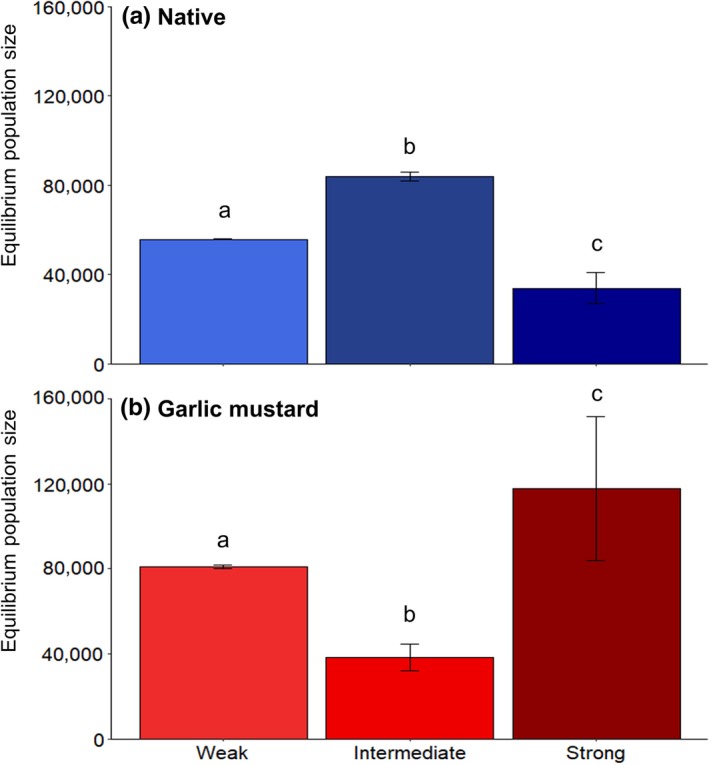
Equilibrium densities of (a) native plant and (b) garlic mustard in model simulations run at three mutualism strengths with an initial density of garlic mustard of 1,000 plants per 400 m^2^ and starting population sizes of 10,000 individuals per 400 m^2^ for both native plant and fungus. Error bars are ±95% confidence intervals. Different letters indicate *p* < 0.05 for Tukey's HSD post hoc comparison following one‐way ANOVA. The native plant was most resistant to invasion at the intermediate‐strength mutualism as shown by its highest final densities at this level of interaction (a), and the fact that density of native plants was >60% only at the intermediate‐strength mutualism (comparison of corresponding densities of native plant and garlic mustard for the three mutualism strengths)

First, for simulations with relatively high initial densities of garlic mustard (1,000 plants per 400 m^2^), the final equilibrium density of the native plant was highest (Figure 3a), and that of garlic mustard was lowest (Figure 3b), at the intermediate mutualism strength. Furthermore, only at intermediate mutualism strength was the final density of the native plant >60% of plant density (Figure 3).

**Figure 4:**
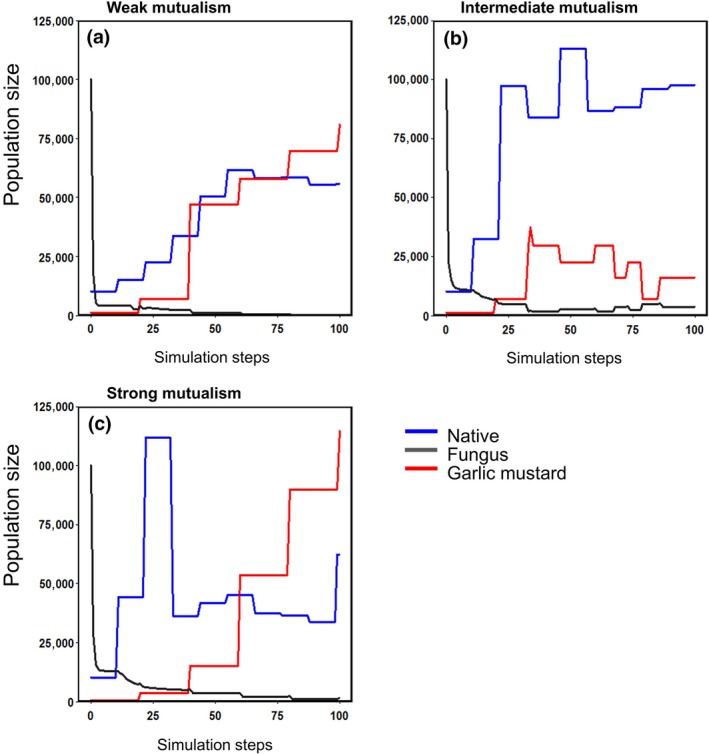
Temporal dynamics of the agents at the initial density of 1,000 garlic mustard plants per 400 m^2^ (a 10‐simulation average). (a) Garlic mustard shows a slow trajectory toward numerical dominance under weak mutualism strength. (b) The native plant remains the most abundant throughout the simulation under the intermediate mutualism. (c) Under strong mutualism strength, the native plant shows an initial large growth in population size but then quickly crashes once the density of fungal hyphae decreases. The native plant and fungus were set at a starting population size of 10,000 individuals per 400 m^2^ for all simulations

Second, with an initial garlic mustard density of 1,000 plants per 400 m^2^, throughout the simulation, the native plant was always more abundant than garlic mustard when the strength of the mutualism was intermediate (Figure 4b); this was not the case for the other mutualism strengths (Figure 4a,c). In addition, the fungus persisted to the end of the simulation only when the strength of the mutualism was intermediate. For the weak mutualism strength, the native plant was more abundant than garlic mustard during the initial steps of the simulation, but garlic mustard eventually became >60% of the plant density as the fungus was eliminated (Figure 4a). Under the strong mutualism, the native plant had a large initial advantage over garlic mustard but rapidly decreased once fungal densities approached zero, and garlic mustard density increased rapidly to over 60% (Figure 4c).

**Figure 5:**
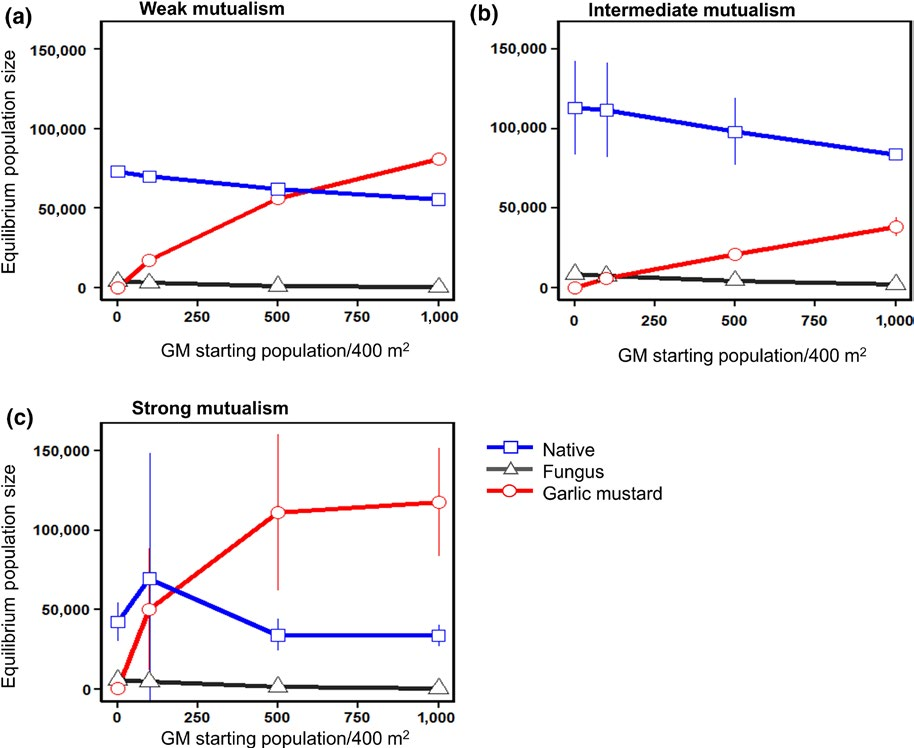
The influence of different initial densities of garlic mustard on equilibrium densities of the three agents under (a) weak, (b) intermediate, and (c) strong mutualism strengths. Over this range of initial densities of garlic mustard, the native plant resists invasion by garlic mustard only under the intermediate‐strength mutualism. Error bars are ±*SE*. GM: garlic mustard

Third, variation in the initial density of garlic mustard (an indication of invasion level) impacted invasion success for weak and strong mutualism strengths (Figure 5a,c), but not for the intermediate‐strength mutualism (Figure 5b). When the mutualism was weak, the final equilibrium density of the native plant declined slightly with increasing initial density of garlic mustard; and garlic mustard became more abundant than the native plant when initial garlic mustard densities were ≥~500 individuals per 400 m^2^ (Figure 5a). For the strong mutualism, the comparable threshold declined to ~100 individuals per 400 m^2^ and the equilibrium density of the native plant declined sharply with increasing initial densities of garlic mustard (Figure 5c). In marked contrast, for the intermediate‐strength mutualism garlic mustard never successfully invaded, that is, its density was always much less than that of the native plant, even when initial densities were 1,000 individuals per 400 m^2^ (Figure 5b).

The fungal response to garlic mustard was similar across mutualism strengths (Figures 4 and 5). Densities declined toward zero the fastest under the weak mutualism strength (Figure 4a). Under intermediate and strong levels (Figure 4b,c), the fungus declined more slowly, though it never reached zero for the intermediate strength. Final fungus density was strongly sensitive to initial garlic mustard density under all mutualism strengths (Figure 5), declining as the final invader density increased. In contrast, there was no such relationship between final densities of the fungus and the native plant (Figure 5).

#### 3.2.2 Initial‐density threshold

**Figure 6:**
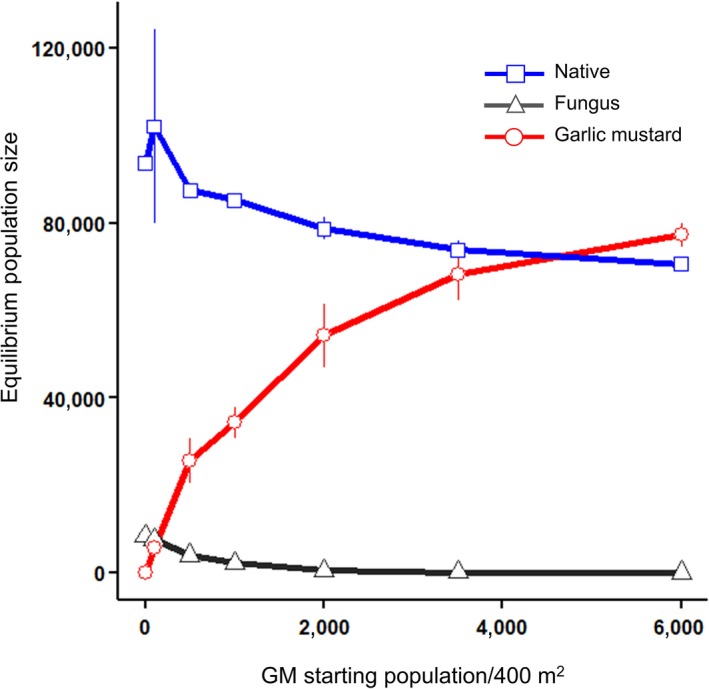
Equilibrium population size of the three agents as a function of initial density of garlic mustard under intermediate mutualism strength, the condition with the maximum resistance to invasions as judged by simulations with initial densities of garlic mustard ≤1,000 plants per 400 m^2^. Error bars are ±*SE*. A starting garlic mustard density of ca. 5,000 plants per 400 m^2 ^appears to be the threshold at which garlic mustard has the competitive advantage over the native plant (a more conservative estimate of the threshold, taking into account the error bars, would be between 3,000 and 6,000 garlic mustard per 400 m^2^)

The preceding results are based upon simulations in which the starting density of the native plant was 10,000 per 400 m^2^; thus, even at the highest initial densities of garlic mustard (1,000 per 400 m^2^), the invasive plant was only ~10% of total plant cover. What would happen if environmental conditions (not included in our model simulations) were to favor garlic mustard for a few generations so that it increased to a higher cover percentage? Is there a threshold beyond which garlic mustard always successfully invades? We addressed this question using the intermediate mutualism strength, the condition most resistant to garlic mustard invasion. A state change occurred once the invader reached ~6,000 garlic mustard plants per 400 m^2^ (Figure 6), where it becomes more abundant than the native plant but still is not considered invasive (represents <60% of plant cover). At ~3,500 garlic mustard plants per 400 m^2^, there was no consistent winner between the native plant and garlic mustard (standard errors overlap, Figure 6). The mycorrhizal fungus went to zero once garlic mustard invasion reached a threshold of 2,000 garlic mustard plants per 400 m^2^ (Figure 6).

#### 3.2.3 Parameters influencing final equilibrium densities

**Figure 7:**
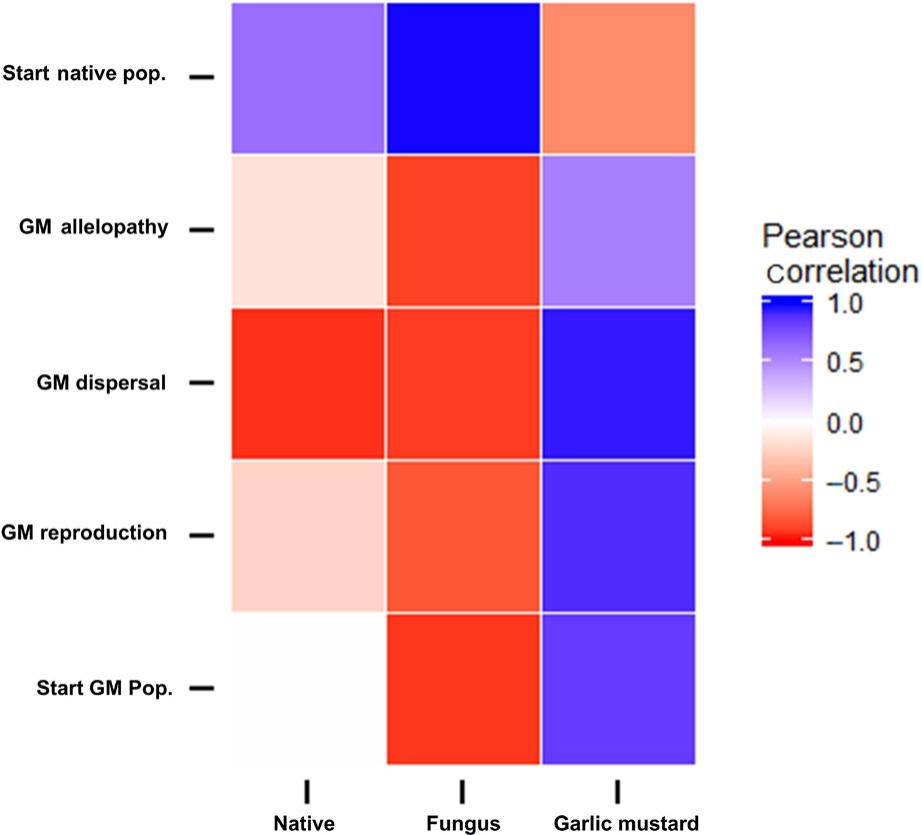
Bivariate heat map showing the relationship between model parameters and output variables (i.e., equilibrium densities of native plant, fungus, and garlic mustard) for the intermediate‐strength mutualism. Blue represents positive Pearson correlations, red denotes negative correlations, and white is no correlation. The darker the color, the stronger the correlation (i.e., closer to 1 or −1). GM allelopathy: garlic mustard allelopathic distance; GM dispersal: garlic mustard dispersal distance; GM reproduction: garlic mustard reproduction per plant; Start GM pop.: size of garlic mustard population at start of simulation; Start Native pop.: size of native population at start of simulation

We investigated which parameters were most influential in determining equilibrium population sizes of the native plant, fungus, and garlic mustard for the intermediate‐strength mutualism (Figure 7). The dispersal ability of garlic mustard had a strong negative effect on the equilibrium density of the native plant, whereas garlic mustard's allelopathy and reproductive output had only a weak negative influence. The initial density of the native plant had a moderately positive impact on its final density. The mycorrhizal fungus was heavily negatively affected by garlic mustard's allelopathy distance, dispersal distance, reproduction, and initial population density. In contrast, the starting population size of the native plant had a strong positive impact on final fungal density. Equilibrium population sizes of garlic mustard were positively affected by its own allelopathy distance, dispersal distance, reproduction, and initial population density. The starting population of the native plant was the only parameter that had a negative impact on the equilibrium population size of garlic mustard (Figure 7).

## 4 DISCUSSION

### 4.1 Effects of mutualism strength on invasion success

Plant–mycorrhizal mutualisms, which are important belowground symbioses in forest ecosystems (Read, Leake, & Perez‐Moreno, 2004), can influence plant and animal populations (Fitter & Garbaye, 1994; Teste et al., 2017). Simulations with our IBM reveal that disruptions to this mutualism can explain invasion success for an allelopathic plant invader. Furthermore, the strength of the plant–mycorrhizal mutualism played a critical role in determining garlic mustard's ability to invade the native plant in our model. Surprisingly, and contrary to our prediction, the mutualism of intermediate strength was the most resistant to garlic mustard invasion, whereas strong and weak mutualisms were both highly susceptible. This pattern was most pronounced at high initial densities of garlic mustard. The intermediate‐strength mutualism afforded *I. capensis* increased fitness when the fungus was present, but the cost of not having fungi was not severe enough to markedly decrease the native's equilibrium population size. Thus, once the native plant established numerical dominance over garlic mustard, the production of offspring was sufficiently high to maintain large population sizes even when the mycorrhizal fungus had decreased to low levels in response to garlic mustard.

Although the native plant and mycorrhizal fungus had higher fitness when the mutualism was strong, garlic mustard was still able to dominate the equilibrium plant community. This outcome was unexpected given that increased fecundity due to the mutualism should have made the native plant a better competitor for space. Early in the simulation, our prediction appeared to be true—the native plant dominated plant density in the first 30–40 time steps. However, once the garlic mustard population was large enough to cause a substantial drop in mycorrhizal fungal densities, the size of the native plant population rapidly declined. Thus, disruption of the symbiosis favored garlic mustard over time since fitness of the native plant without the obligate mutualist was too low to overcome competition for space with the invader. This finding supports a body of literature that shows members of obligate (i.e., strong) mutualisms are more susceptible to environmental changes (Takimoto & Suzuki, 2016). For example, obligate plant‐pollinator mutualists are constrained from switching partners, rendering them susceptible to extinction due to partner loss (Pellmyr, Thompson, Brown, & Harrison, 1996; Sachs & Simms, 2006). Despite the fact that in our model, in the absence of an invader, high levels of mutualism strength had a higher fitness than intermediate levels, a strong plant–mycorrhizal mutualism is more likely to become dominated by an allelopathic plant invader because the invader eventually has a too ‐ strong negative effect on the relative fitness of the symbiotic relationship.

Weak plant–mycorrhizal interactions were also more susceptible to garlic mustard invasion compared to intermediate symbiotic strengths. Unlike the situation for obligate mutualisms, under weak facultative mutualisms, the presence of the fungus had only a minor effect on plant growth and reproduction; nevertheless, garlic mustard still became a numerical dominant under this condition. Two factors can explain this result. First, the fitness of both plant and fungus was not sufficient to outcompete garlic mustard, and because there is little exchange of nutrients/resources between the native plant and the fungus, the mutualism had a negligible impact on competitive ability. This lack of resource exchange effectively lowered the fitness of the native plant, leading to garlic mustard's dominance. Second, once garlic mustard establishes in the model, the native plant does not have a secondary trait (such as high seed production or fast generation times) that can overcome the fitness advantage of the plant invader. Thus, as garlic mustard colonizes more patches, the intrinsically low fitness of the native plant leads it toward extinction.

### 4.2 Invasion threshold for garlic mustard

Even though the plant–mycorrhizal mutualism of intermediate strength was least impacted by garlic mustard invasion under certain combinations of parameters, the native plant did not always dominate garlic mustard numerically. If an environmental condition not explicitly included in our IBM were to allow garlic mustard to reach a threshold of ca. 6,000 plants per 400 m^2^ (perhaps even lower, given the size of the SE's for some densities), our model predicts that garlic mustard would become numerically dominant, and perhaps could limit the abundance of the native plant even further if the simulation were to continue. This finding indicates that initial site conditions and non‐native plant densities could have profound effects on the success of a plant invader. Several studies have suggested that a habitat must be degraded or substantially altered before a non‐native plant can become established as an invasive (Dassonville et al., 2008; Davis, Grime, & Thompson, 2000; Suding et al., 2013). *Carduus pycnocephalus *(calflora), for example, became a more aggressive invasive herbaceous plant when it was sown under conditions where native mycorrhizal fungi were suppressed (Vogelsang & Bever, 2009). In a situation where the system has been previously degraded, an invasive plant like garlic mustard can establish itself and further erode the system through its persistent allelopathy, though for garlic mustard, the effects appear most pronounced in early invasions. Our IBM indicates that even native plants with substantial but not obligate symbioses with mycorrhizal fungi will have difficulty resisting invasion by plants such as garlic mustard if environmental conditions give invasives an initially large numerical advantage.

### 4.3 FUTURE RESEARCH DIRECTIONS AND CONCLUSIONS

Our IBM indicates that the strength of the mutualism between native plants and mycorrhizal fungi may be an important factor influencing invasion success of an allelopathic plant invader such as garlic mustard. In particular, we show that plant–mycorrhizal mutualisms that are either weak or strong are less resistant to invasion than intermediate‐strength mutualisms. Though our model results may provide practical applications for identifying native plants at risk of displacement by an allelopathic plant invader, empirical research is needed to validate if these patterns are true in nature. One such study might be to establish, in a greenhouse, a gradient of plant communities with known dependencies on mycorrhizal fungi (i.e., a plant community that is highly dependent upon mycorrhizal mutualisms, a community with intermediate dependencies, and so forth). Once this gradient of plant communities is established, experimentally introducing allelopathic plant invaders would test which communities are most resistant to invasion. Another study might be to identify a plant with plastic responses to mycorrhizal fungi; for example, a plant that is less dependent on fungi in high‐nutrient conditions but more dependent in nutrient‐poor conditions. Performing experimental introductions with allelopathic invaders across different conditions of mycorrhizal dependencies would shed light on how mutualism strengths can mediate invasion success in nature. Given that many other environmental factors, such as natural enemies, may influence how plants interact with mycorrhizal fungi, conducting the above studies as field experiments would provide the strongest tests of the predictions of our IBM.

Due to the increasing disruptions to ecosystems by invasive plants, this research provides timely insights into how an allelopathic invasive plant may successfully invade native plant communities. We show that plant–mycorrhizal mutualisms of intermediate strengths are most resistant to garlic mustard invasion. We also show there might be invasion thresholds even for the plants most resistant to plant invasion. Thus, forest plants that are of conservation interest should be tested for their reliance upon mycorrhizal fungi, as our model suggests their level of dependence on this mutualism will likely determine how vulnerable they are to displacement by an allelopathic invader. In addition, forest ecosystems that have experienced degradation that weakens existing plant–mycorrhizal mutualisms, or that gives a sudden numerical advantage to an allelopathic invader, are likely to be more susceptible to invasion. Hence, we call for future field studies to evaluate the “Intermediate Mutualist‐Strength Hypothesis” for plant invaders that negatively impact the mutualism between native plants and mycorrhizal fungi.

## CONFLICT OF INTEREST

None declared.

## AUTHOR CONTRIBUTION

MM and MZ developed the initial ideas, designed, and tested the validity and robustness of the individual‐based model; DH provided intellectual guidance and helped determine what inputs the model should include; MM analyzed the data; MM led the writing of the manuscript. All authors contributed substantially to the writing and gave final approval for submission.

## Data Availability

The NetLogo model and script are uploaded as an online supporting document.
