# Supplementary material for: The role of plant–mycorrhizal mutualisms in deterring plant invasions: Insights from an individual‐based model
Source: Ecol Evol. 2019 Jan 28;9(4):2018–30. doi: 10.1002/ece3.4892 (PMC6392346; doi:10.1002/ece3.4892)
Supplement: Supplementary file 1 [file ECE3-9-2018-s001.docx]

**The role of plant-mycorrhizal mutualisms in deterring plant invasions: Insights from an individual-based model**

Matthew A. McCary^1, 2, 4*^, Moira Zellner^2, 3^, and David H. Wise^1, 2^

^1^ Department of Biological Sciences, University of Illinois, Chicago, IL 60607, USA

^2^ Institute for Environmental Science and Policy, University of Illinois, Chicago, IL 60612, USA

^3^ College of Urban Planning and Public Affairs, University of Illinois, 412 South Peoria Street, Chicago, Illinois 60607 USA

^4^ Department of Entomology, University of Wisconsin, Madison WI 53706, USA (Present Address)

**The model description below follows the ODD protocol (overview, design concepts, details) for describing individual-based models (Grimm et al. 2010).**

*Purpose*

This model examines how plant-mycorrhizal mutualism strengths influence invasiveness for an allelopathic invader, *Alliaria petiolata* (garlic mustard). Two questions were investigated: (1) How might the strength of the mutualism between a native plant (*Impatiens capensis*) and a mycorrhizal fungus affect the plant’s resistance to garlic mustard invasion? (2) Can there be a non-linear relationship between initial garlic mustard density and establishment?

*Entities, state variables, and scale*

We constructed a spatially explicit individual-based model in NetLogo version 5.3.1. To mitigate edge effects (Railsback & Grimm 2012), we designed a torus world that was a 200 × 200 square grid representing patches of soil. Each patch (1 dm^2^) served as spaces that plants and fungi could colonized, as well as served as the fungus-root interface where nutrients are exchanged. There were three agents in this model: (1) a plant that is representative of a native annual of North America (*Impatiens* *capensis* [hereafter referred to as the “native plant”]); (2) a “generic” mycorrhizal fungus that forms a mutualistic association with the native plant; and (3) a non-native invasive plant (garlic mustard). The initial units and state variables for each agent are provided in Table S1 below.

Native plant – The first agent of this model simulates the characteristics of *I. capensis*, which is an annual herb native to North American forests. Garlic mustard and *I. capensis* are often found in similar habitats. Furthermore, *I. capensis* is documented to form mutualistic interactions with mycorrhizal fungi (Cipollini et al. 2008). *I. capensis* is a dehiscence ballistic disperser (Hayashi et al. 2009), meaning the seed pod explodes to release seeds that travel a short distance (3 - 5 dm; Schmitt et al. 1985).

Mycorrhizal fungus – The second agent has fungal nodes and associated links (i.e. extraradical mycelium) to mimic the growth of a mycorrhizal fungus, which forms a mutualistic interaction with the native plant. Mycorrhizal fungi form symbiotic relationships with the roots of most plant species: the plants benefit from increased absorption of water and nutrients, whereas the mycorrhizal fungi get ***C*** in return. In this model, the mycorrhizal fungus grows and ‘sprouts’ new fungal nodes faster in the presence of a native plant. Similarly, the native plant grows and reproduces more offspring when the fungus is present. Here, we manipulate the rate of nutrient exchange to alter the strength of the mutualism (e.g. high rates of nutrient exchange = stronger mutualism). The fungus also has the ability to re-allocate ***C*** to other depleted mycorrhizae that are deficient in nutrients.

Garlic mustard – The third agent of this model is the invasive, non-native garlic mustard – a biennial, shade-tolerant, and non-mycorrhizal herb that has invaded forests in North America (Anderson et al. 1996; Nuzzo 1999). Garlic mustard produces secondary compounds, such as glucosinolates, that are toxic to micro-organisms. Glucosinolates in garlic mustard are known to disrupt the plant-mycorrhizal mutualism (Cipollini 2002; Stinson et al. 2006). Glucosinolates released from garlic mustard tissue reduce both arbuscular (Roberts & Anderson 2001; Stinson et al. 2006) and ectomycorrhizal fungi (Wolfe et al. 2008). In this model, garlic mustard is programmed to kill mycorrhizal fungi. In this model we defined invasion success as garlic mustard representing ≥60% of the plant population at the end of the simulation.

Patch environment – The soil patches, i.e. the model environment, represent areas that either the native plant, fungus, or garlic mustard could colonize. Two plants (same or different species) cannot colonize the same patch; however, the fungus can colonize a patch even when there is another plant or fungus established. The patch is also where the exchange of nutrients can occur between the mycorrhizal fungus and native plants’ roots. When a fungus is present, the native plant will release ***C*** for the fungus to uptake, and also allows for increased nutrient absorption by the native plant. Garlic mustard does not release photosynthetic ***C*** in to the patch because it does not form a mutualistic association with mycorrhizal fungi. Initial photosynthetic ***C*** values across the model environment are randomly selected from a normal distribution to allow for fungal nodes to propagate at the start of the simulations.

We simulated the introduction of garlic mustard under the following conditions (see Table S1 and Table 1 in the main text): (i) weak, intermediate, and strong levels of the plant-mycorrhizal mutualism [i.e. low versus high levels of nutrient exchange and reproductive outputs]; and (ii) different initial numbers of the invasive plant. For each scenario we documented, after 100 time steps, how many fungal nodes were present and the population sizes of the native plant and garlic mustard. We also collected population sizes for each time step.

*Process overview and scheduling*

Following initialization, all individual plants are randomly distributed in the model world. Competition for space between the native and non-native plants then takes place within each time step. An annual native plant that acquires a symbiosis with a nearby mycorrhizal node will receive more nutrients over its life span (10 time steps). When nutrient exchange is high between the native plant and fungus, the interaction between the two species acts as a mutualism. In contrast, in patches where the invasive plant is present, the mycorrhizal fungus is ‘killed’ within a given radius and the invasive plant gains a fitness advantage. The invasive plant, a biennial, reproduces after 20 time steps and “passively” acquires energy during each step (i.e. from photosynthesis not associated with the fungus).

*Design concepts*

Basic principles – The theory of “Mutualism Disruption” (Hale et al. 2011) postulates that invasive species gain a competitive advantage over native species by causing a disruption to a key mutualism. This model uses this conceptual framework to examine how plant-mycorrhizal mutualism strengths can mediate invasion success of garlic mustard in North American forests.

Emergence – The results from the model indicate that intermediate localized mutualism strengths between the native plant and the mycorrhizal fungus lead to a resistance to invasion by garlic mustard at the population level. We also observed that garlic mustard outcompetes the native plant once it reaches high initial densities regardless of the mutualism strength.

Adaptation – None.

Objectives – None.

Learning prediction – None.

Sensing – The mycorrhizal nodes are capable of ‘sensing’ their environment when their carbon is low in order to search for nearby fungi with available carbon.

Interaction – The native plant and garlic mustard interact via competition for space, which is mediated by the presence of a mycorrhizal fungus.

Stochasticity – Initial photosynthetic ***C*** values across the patch environment are randomly selected from a normal distribution to allow for fungal nodes to propagate at the start of the simulations. Also, the radius in which native or invasive plants can disperse their offspring is a stochastic property. Here, plants can produce offspring on any open patch that falls within a specific radius of the mother plant. We also modeled a stochastic process associated with reproduction. Instead of fixed offspring a given plant can have, the amount of offspring is randomly selected from a normal distribution within a bounded interval (e.g., when garlic mustard reproduction is set to 15, it can reproduce anything between 1 and 15).

Observation – Population size of each plant and abundances of mycorrhizal nodes are the main observations.

*Initialization*

Before initialization, each patch in the model is randomly assigned a photosynthetic ***C*** value in which a native or invasive plant can be present. There are also mycorrhizal nodes that are randomly distributed within the model environment. Each plant has a pre-defined initial density, reproductive capacity and dispersal ability; garlic mustard has the additional trait of being able to kill mycorrhizal fungi within a given radius. After initialization the agents compete for space and resources.

*Input*

This model does not incorporate input data.

*Sub-models*

Native plant-mycorrhizal mutualism – The first sub-model simulates the mutualistic interaction between a native plant and a mycorrhizal fungus. Here, the number of offspring produced by the native plant is heavily influenced by the presence/absence of the mycorrhizal fungus, i.e. when the mycorrhizal fungus is present, the reproductive output of the native plant is higher due to increased rates of nutrient acquisition (in NetLogo nutrients are defined as generic units of ‘energy’). Specifically, we modeled three *high* rates of acquisition when the fungus is present (***E*** = 0.45, 0.75, or 1 energy units time step^–1^), and three *low* rates when the fungus is absent (***e*** = 0.01, 0.3, or 0.4 energy units time step^-1^). At the end its life cycle, the native plant will either produce high or low offspring per plant—a reproductive output that is directly linked to the amount of energy acquired over its life span. A native plant with a fungal mutualism will produce more offspring; a native plant without a fungal interaction will produce fewer. Thus, the fitness of the native plant is reduced when the mycorrhizal fungus is absent, which is a well-documented pattern. We programmed for the native plant to set seed and die after one growing season (i.e. ten time steps in the model [representing ~100 days]. In the model native plants can reproduce up to a certain distance (4 patch units corresponding to 4 dm), which is done using a random draw between the 1 and 4 distance units. The direction in which a native plant can produce offspring is also random.

To help service the mycorrhizal fungus, the native plant provides ***C*** to the fungus only when it is available. We specifically modeled the native plant to release a pre-defined unit of ***C*** to the fungus during each time step when a fungus is present. If nutrient acquisition is high for the native plant due to the mutualism, the plant will release more photosynthetic ***C*** to the fungus. Under conditions where fungi are present, ***C*** will be released by the native plant at units of 4.5, 9, and 15 µmol ***C*** time^-1^ (***C_r_***). When fungus is absent, it will release no ***C***.

Studies to date have estimated that mycorrhizal fungi metabolize available ***C*** from plant roots at rates up to 20-30% (Hobbie et al. 2006).We simulated ***C*** to be metabolized within that range of values (***C_m_*** = 1.5 µmol C time step^-1^ (33%), 2.5 (28%), and 4 (26)%). The fungus starts as a spore (i.e. a NetLogo node) and has the capacity to grow hyphae (i.e. links) in the direction of patches with high photosynthetic ***C*** values. The nodes grow until a certain size is reached (0.3 mm), which is dependent on photosynthetic ***C*** availability, and then new connections are made with high photosynthetic ***C*** values (i.e. ≥ 0.5 ***C*** concentration). The growth of the nodes depends on the strength of the mutualism: slow rates of fungal growth under weak mutualisms (0.01 mm time step ^-1^), medium growth under intermediate mutualisms (0.15 mm time step ^-1^), and fast growth under strong mutualism (up to 0.3 mm time step^-1^). If the available photosynthetic ***C*** falls to zero because there is no native plant is present, the fungal node can receive ***C*** from a nearby neighbor. However, once the available photosynthetic ***C*** falls to zero and no other neighbor can help, that fungal node will die.

Garlic mustard invasion – The second sub-model is the introduction of the invasive garlic mustard. Garlic mustard is simulated to invade the system and reduce the abundance of mycorrhizal nodes via allelochemicals (i.e. releasing of toxic chemicals into the environment), thereby indirectly affecting the native plant population. Specifically, when garlic mustard invades the simulation, nodes and mycorrhizal hyphae instantly die within a radius that is set prior to model initiation (***A*** = 2.5 dm). Additionally, we have simulated garlic mustard to produce new offspring within a range of distances from the mother plant (***D*** = 3 dm). The number of new offspring an invasive plant can produce ranges up to 15 offspring per plant (***R***). Here, a random number between 1 and 15 is generated, which then serves as the number a given garlic mustard plant can reproduce. Like the native plant, the direction in which a garlic mustard can produce offspring is random.

The garlic mustard reproduces after 20 time steps (the native plant, an annual, dies after 10 time steps; thus the 20-step threshold for garlic mustard reflects the fact that in nature garlic mustard is biennial). Garlic mustard, which forms no mutualistic interactions with mycorrhizal fungi, does not release photosynthetic ***C*** and does not exchange nutrients with fungi (Anderson et al. 1996). We simulated garlic mustard invasion (***I***) under the following initial conditions: 1 individual per 400 m^2^ (0% plant cover), 100 individuals per 400 m (1% plant cover), 500 per 400 m^2^ (5% plant cover), and 1000 per 400 m^2^ (~10% plant cover). These values were chosen to represent early stages of plant invasion (Anderson et al. 1996; Nuzzo 1999).

**Table S1.** State variables and initialization in the individual-based model. Values that are estimated from published articles are filled in where applicable. POM = Process Oriented Modeling.

| **Variables** | **Description** | **Values** | **References** |
| --- | --- | --- | --- |
| *Native Plants* |  |  |  |
| ***E*** | Photosynthetic rate w/ fungus present | 0.45, 0.75, 1 [energy units time step^-1^] | POM |
| ***e*** | Photosynthetic rate w/o fungus present | 0.01, 0.3, 0.4 [energy units time step^-1^] | POM |
| ***C_r_*** | Carbon molecules released w/ fungus | 4.5, 9, 15 [C units time step^-1^] | POM |
|  |  |  |  |
| *Fungi* |  |  |  |
| ***F*** | Spore fungal growth rate w/ native plant | 0.01, 0.15, 0.3 [g time step^-1^] | POM |
| ***C_m_*** | Carbon metabolization | 1.5, 2.5, 4 of plant carbon release [µmol C time step^-1^] | Hobbie 2006 |
|  |  |  |  |
| *Garlic mustard* |  |  |  |
| ***I*** | Starting no. invasive plants | 1, 100, 500, 1000 [400 m^-2^] | Anderson et al. 1996 |
| ***R*** | No. offspring an invasive can produce | ≤ 15 per plant | Anderson et al. 1996 |
| ***D*** | Distance an invasive can produce offspring | 3 dm | Nuzzo 1999 |
| **A** | Patch radius garlic mustard can suppress fungi | 2.5 dm | Wolfe et al. 2008 |
|  |  |  |  |
| *Patches* |  |  |  |
| ***C*** | Carbon value for patch | ≤ 4, ≤ 8, ≤ 9 | POM |
|  |  |  |  |
| *Initialization* |  |  |  |
| Native density | Starting no. *native* plants | 10000 [400 m^-2^] | Steets & Ashman 2010 |
| Native dispersal | Patch distance a *native* plant can sprout new plants | 1 [dm] | POM |
| Native high | No. offspring produced when energy is high | 3 [dm^-2^] | POM |
| Native low | No. offspring produced when energy is low | 1 [dm^-2^] | POM |
| Invasive energy | Photosynthetic carbon acquired | 1 [mol time step^-1^] | POM |
| Fungal node | Mycorrhizal node initial abundance | 10000 [400 m^-2^] | POM |
| Node size | Maximal node biomass | 0.3 [mm] | POM |
| Hyphae | Maximal extraradical hyphae size (links) | 0.3 [mg] | POM |
|  |  |  |  |

**REFERENCES**

Anderson, R. C., Dhillion, S. S. & Kelley, T. M. (1996) Aspects of the ecology of an invasive plant, garlic mustard (*Alliaria petiolata*), in central Illinois. *Restoration Ecology,* **4,** 181-191.

Cipollini, D. (2002) Variation in the expression of chemical defenses in *Alliaria petiolata* (Brassicaceae) in the field and common garden. *American Journal of Botany,* **89,** 1422-1430.

Cipollini, K. A., McClain, G. Y. & Cipollini, D. (2008) Separating above-and belowground effects of *Alliaria petiolata* and *Lonicera maackii* on the performance of Impatiens capensis. *The American Midland Naturalist*, **160**, 117-128.

Grimm, V., Berger, U., DeAngelis, D. L., Polhill, J. G., Giske, J. & Railsback, S. F. (2010) The ODD protocol: a review and first update. *Ecological modelling,* **221,** 2760-2768.

Hale, A. N., Tonsor, S. J. & Kalisz, S. (2011) Testing the mutualism disruption hypothesis: physiological mechanisms for invasion of intact perennial plant communities. *Ecosphere,* **2,** 1-15.

Hayashi, M., Feilich, K. L. & Ellerby, D. J. (2009) The mechanics of explosive seed dispersal in orange jewelweed (*Impatiens capensis*). *Journal of experimental botany,* **60,** 2045-2053.

Hobbie, E. A. (2006) Carbon allocation to ectomycorrhizal fungi correlates with belowground allocation in culture studies. *Ecology,* **87,** 563-569.

Nuzzo, V. (1999) Invasion pattern of herb garlic mustard (*Alliaria petiolata*) in high quality forests. *Biological Invasions,* **1,** 169-179.

Railsback, S. F. & Grimm, V. (2012) *Agent-based and individual-based modeling: a practical introduction.* Princeton University Press, New Jersey, USA.

Roberts, K. J. & Anderson, R. C. (2001) Effect of garlic mustard [*Alliaria petiolata* (Beib. Cavara & Grande)] extracts on plants and arbuscular mycorrhizal (AM) fungi. *The American Midland Naturalist,* **146,** 146-152.

Schmitt, J., Ehrhardt, D. & Swartz, D. (1985) Differential dispersal of self-fertilized and outcrossed progeny in jewelweed (*Impatiens capensis*). *The American Naturalist,* **126,** 570-575.

Steets, J. & Ashman, T. L. (2010) Maternal effects of herbivory in *Impatiens capensis*. *International journal of plant sciences,* **171,** 509-518.

Stinson, K. A., Campbell, S. A., Powell, J. R., Wolfe, B. E., Callaway, R. M., Thelen, G. C., Hallett, S. G., Prati, D. & Klironomos, J. N. (2006) Invasive plant suppresses the growth of native tree seedlings by disrupting belowground mutualisms. *PLoS biology,* **4,** 727.

Wolfe, B. E., Rodgers, V. L., Stinson, K. A. & Pringle, A. (2008) The invasive plant *Alliaria petiolata* (garlic mustard) inhibits ectomycorrhizal fungi in its introduced range. *Journal of Ecology,* **96,** 777-783.
